# Migraine

**Published:** 1907-08-24

**Authors:** Purves Stewart


### MIGRAINE.

Being an Abstract of a Lecture thereon by PURVES STEWART, M.A., M.D.Edin., F.R.C.P.London.

The aura in migraine is a subjective sensation of some kind, and the commonest is the visual. It is generally hemianopic in its distribution, occupying and confined to one half of the visual field. The visual phenomena are generally on the side opposite to the headache. The patient begins to notice a sort of blurring of his vision, which may increase to actual blindness, so that he sees nothing on that side; or he may have subjective colour sensations such as a luminous spectrum or a very fine zigzag fluctuating pattern, enclosing a blind area, which soon increases in size and spreads out over half the visual field. In rare cases the upper half of the field of vision is lost, or there may be a central scotoma.

### The Aura.

Among the other common auræ is a tingling sensation spreading up one hand or one arm to the face, lips, mouth, or tongue. That is fairly common, and is followed by pain on the other side of the head. If this tingling sensation is on the right side of the body and spreads to the face, it may be accompanied by a slight degree of aphasia.

An eminent "migrainist" lecturer of my acquaintance knows he is going to have an attack when he is lecturing because he begins to use the wrong words. He thereupon promptly stops his lecture and makes preparations for the attack. The tingling arm is clumsy and weak. If the tingling is in the tongue it may be difficult for the patient to articulate his words. We have therefore to diagnose cases of migraine from epilepsy, especially Jacksonian epilepsy. There may be auræ of smell or taste, and rarely of hearing, just as in the epileptic fit.

### Later Symptoms—Headache.

After the aura comes the headache and its associated phenomena. The headache is generally one-sided, either exclusively or preponderatingly. The site of maximum pain is generally over the eye, and from that fact the old English name "brow ague" is given.

The patient is abnormally sensitive to stimuli such as loud sounds and bright light, and, if left to himself, goes to the quietest room in the house and pulls down the blinds.

Some people during an attack of headache have flushed features, others have pallor; some start with pallor, and finish with flushing. Some do not change colour at all. The pupil on the affected side is sometimes contracted, sometimes dilated, and sometimes unchanged, and its condition is of no real diagnostic value. The patient has a violent distaste for food. As the headache continues, he develops nausea, and this nausea culminates in vomiting, and the patient then knows that the end is in sight. The vomited material consists largely of mucus or of bile. That is why this is called a "bilious" headache, though the attack has nothing to do with the liver. After vomiting, the headache generally comes to an end, the patient falls asleep, and wakes up quite well. In some cases the vomiting may be replaced by a critical diarrhœa, sweating, or lacrymation.

In a moderately severe case the headache lasts about twelve hours. In other cases it may be prolonged for days, or for a week—a most distressing and fortunately rare condition called the *status hemicranicus*. Sometimes the patient is partially aphasic the whole time, and gross intracranial disease is frequently suspected. In about half the cases the visual aura and primary symptoms are present. Others have no headache at all, only the aura followed by vomiting.

### Commencement and Course.

Migraine generally begins in childhood, and recurs in more or less regular attacks, say, once a fortnight or once a month; it attains its maximum severity during a period of life when the greatest efforts have to be performed. It is most troublesome during youth and middle age. After the age of fifty in both sexes it tends to diminish, and ultimately to disappear. Moebius has clearly shown that in nine cases out of ten migraine is a hereditary disease. We can sometimes trace quite definitely certain exciting causes which precipitate an attack in hereditarily predisposed patients; the commonest are mental strain and excitement, alcoholic excesses, and worry. There are other less important factors, such as gastro-intestinal disorders, constipation, menstruation, which sometimes precipitate an attack. Errors of refraction have been emphasised by Gould, of Philadelphia, as exciting causes, but their frequency is probably exaggerated. Most patients learn by bitter experience what particular exciting cause brings on their attack.

### Diagnosis.

There is no difficulty in typical cases of migraine. The difficulty is with the abortive and incomplete cases. An important point to remember in diagnosis is the complete immunity of the patient between the paroxysms of the attacks, and this one fact marks off migraine from a very large number of other pains which may be somewhat similar in distribution.

### Differential Diagnosis.

Frontal-sinus suppuration does not cause paroxysmal pain, but is continuous. The pain of glaucoma may be confused with migraine, but here again the paroxysmal element assists diagnosis. The headache of syphilitic periostitis may be very intense, and even cause vomiting. The headache and vomiting of uræmia and the gastric crises of tabes can only be confused by the very superficial observer. Intracranial tumour sometimes simulates migraine, but in cerebral tumours we generally have some other physical sign, such as optic neuritis or paralysis. It is essential to bear in mind that certain tumours, especially of the occipital region, may produce migrainous visual phenomena, and until neuritis develops it may be impossible to say whether or not the patient has anything more than migraine. Most cases of true migraine come on in childhood, tumours at a later period of life.

Migraine in a number of cases is associated with epilepsy, and it may alternate with epileptic fits in some patients. We may have to diagnose between a migrainous attack and an epileptic fit, and this is sometimes a matter of considerable difficulty. The main thing to remember is that true epilepsy is associated with unconsciousness, whereas migraine is not. In Jacksonian fits, which may not be accompanied by unconsciousness, there is sometimes difficulty in diagnosing between the two conditions.

### Pathology.

The pathology is much in dispute. There can be no doubt that it is a brain disease, but no gross anatomical lesions have been described in migrainous patients. It may be due to some lesion of the cortex in the occipital lobe, which would explain hemianopic symptoms. The pain is most likely due to irritation of the meninges, because brain disease itself does not cause pain as a rule until the membranes are affected. The vomiting is undoubtedly cerebral in origin. The great difficulty is to explain the recurrent paroxysms. To talk about "brain storms" does not advance our knowledge in the least. Some authors have written long treatises on the *rôle* of the cervical sympathetic in the production of dilated pupil and flushed face. But patients with gross disease of the sympathetic do not have migrainous attacks, moreover, this explanation throws no light upon the paroxysmal character of the disease. Another theory is that of recurrent toxæmia, and it is supposed that morbid products which give rise to pain accumulate during the intervals. This does not explain why the disease is only one-sided, nor why it tends to get well with advancing years, when most other toxic diseases become aggravated. Perhaps the most ingenious theory which has been set forth is Spitzer's theory—that the primary lesion is a congenital stenosis of the foramen of Monro. This foramen conects the lateral and ventricles. The choroid plexuses of the lateral ventricles are continuous with each other and with those of the third ventricle through the foramen of Monro. If for any reason the choroid plexus closes up one or other limb of the foramen, all the phenomena of migraine follow, owing to over-distension of the corresponding lateral ventricle by the retained cerebro-spinal fluid, which cannot get out into the spinal canal. The distended ventricle stimulates the visual cortex and may produce the tinglings and the various other sensory auræ; later, by pressing the meninges, it produces headache. Finally the pressure becomes so great that the choroid plexus prolapses and permits the escape of the fluid into the third ventricle, when the paroxysm comes to an end. The foramen is temporarily dilated and only slowly contracts to its original size. This theory explains the recurrence of the symptoms, and also why patients tend to get better in old age.

### Treatment.

We have to recognise that there is no one line of treatment applicable in every case, but there are a few general maxims which may be borne in mind. Migraine is a strongly hereditary disease. It is impracticable, however, to suggest that migrainous patients should not marry. Certain hygienic rules should be kept. Patients ought to avoid a sedentary occupation, especially if it is associated with a stuffy atmosphere. City offices are bad for migrainous patients, who should also be teetotalers. Excess, whether in food, tobacco, or in any other respect, should be avoided. Worry and all forms of excitement are bad. The gastro-intestinal functions should be regulated, the pelvic organs investigated in female patients, and errors of refraction set right.

### Drugs.

During the actual attack the patient usually retires to a dark room and lies down, to avoid noise and bright light. No single drug is a panacea, but that which in one form or other relieves the majority of cases is caffeine. The most convenient vehicle of administration is good black coffee. Various analgesic drugs are useful, the best being phenacetin, acetanilide, salicylic acid, or caffeine citrate combined with phenacetin and one of the bromides. Sometimes the same drug will succeed during one attack and fail the next time; for that reason we must be prepared to change our remedies. An excellent thing to try in many cases, especially if the hands and feet are cold, is to put the patient up to his neck in a hot bath. This relieves the brain hyperæmia, and can be assisted by cold applications to the head.

